# Serum Sphingolipids Aiding the Diagnosis of Adult HIV-Negative Patients with *Talaromyces marneffei* Infection

**DOI:** 10.3389/fcimb.2021.701913

**Published:** 2021-06-28

**Authors:** Zheng-Tu Li, Lee-Fong Yau, Ye Qiu, Shao-Qiang Li, Yang-Qing Zhan, Wai-Him Chan, Zhao-Ming Chen, Zhun Li, Yongming Li, Ye Lin, Jing Cheng, Jian-Quan Zhang, Zhi-Hong Jiang, Jing-Rong Wang, Feng Ye

**Affiliations:** ^1^ State Key Laboratory of Respiratory Disease, National Clinical Research Center for Respiratory Disease, Guangzhou Institute of Respiratory Health, The First Affiliated Hospital of Guangzhou Medical University, National Center for Respiratory Medicine, Guangzhou, China; ^2^ State Key Laboratory of Quality Research in Chinese Medicine, Macau Institute for Applied Research in Medicine and Health, Macau University of Science and Technology, Taipa, Macau; ^3^ Department of Comprehensive Internal Medicine, The Affiliated Tumor Hospital of Guangxi Medical University, Nanning, China; ^4^ Department of Respiratory and Critical Medicine, The Eighth Affiliated Hospital, Sun Yat-Sen University, Shenzhen, China

**Keywords:** *Talaromyces marneffei* infection, sphingolipidomics, sphinganine, diagnosis, serum biomarker

## Abstract

Increasing attention has been directed to *Talaromyces marneffei* (*T. marneffei*) infection in HIV-negative patients due to its high mortality rate. However, nonspecific symptoms and biological characteristics similar to those of other common pathogenic fungi complicate the rapid and accurate diagnosis of *T. marneffei* infection. Sphingolipids (SPLs) are bioactive lipids involved in the regulation of various physiological and pathological processes and have been identified as serum biomarkers for several diseases. This study employed a sphingolipidomic approach established in our previous work to explore the use of serum SPLs in the diagnosis of HIV-negative patients with *T. marneffei* infection. Additional clinical cohorts of patients infected with other microorganisms were also recruited. We found that sphinganine (Sa) (d16:0) exhibited obvious depletion after infection; moreover, its level in patients with *T. marneffei* infection was significantly lower than that in patients infected with other microorganisms. Therefore, Sa (d16:0) was considered a specific diagnostic biomarker for *T. marneffei* infection, and 302.71 nM was selected as the optimal cutoff value with a diagnostic sensitivity of 87.5% and specificity of 100%. These results suggested that determination of serum Sa (d16:0) levels can be used as a new alternative tool for the rapid diagnosis of *T. marneffei* infection in HIV-negative patients.

## Introduction

*Talaromyces marneffei* (*T. marneffei*) is a dimorphic fungus causing life-threatening opportunistic infections that is endemic in southern China, southeastern Asia, and northeastern India, and it was previously considered the agent of a common infectious disease complication of HIV patients (Le et al., 2017; Guo et al., 2020). However, it was found in recent reviews that the morbidity due to *T. marneffei* infection in HIV-negative patients has been increasing (Hu et al., 2013; Chan et al., 2016), which is associated with changes in susceptibility factors. In addition to HIV patients, those who have a chronic underlying disease, including hematologic malignancy, diabetes, tuberculosis, systemic lupus erythematosus (Luo et al., 2011), bone marrow or organ transplantation (He et al., 2020), immunosuppressant use (Lin et al., 2010), or elevated anti-IFN-γ autoantibody levels (Guo et al., 2020), and even healthy people (Lee et al., 2012) have become susceptible to *T. marneffei* infection. Furthermore, once HIV-negative patients are infected, they are difficult to treat, and their fatality rate is significantly higher than that of HIV patients (29.4% vs 20.7%) (Kawila et al., 2013; Chan et al., 2016). Therefore, it is necessary to direct more attention to HIV-negative patients infected with *T. marneffei*.

Similar to other pulmonary mycoses, histopathology or a positive culture of *T. marneffei* with sterile body fluids are the gold standard methods to diagnose *T. marneffei* infection. Nevertheless, these diagnostic methods are typically time consuming and laborious, and the possible invasiveness and complications of these methods also limit their application by physicians. Hence, the exploitation of serum biomarkers has become an emerging significant strategy for early diagnosis. To date, definitive diagnostic serum biomarkers for certain pulmonary mycoses, such as pulmonary aspergillus disease or candidiasis, have been discovered (Donnelly et al., 2019); however, prospective serum biomarkers capable of diagnosing *T. marneffei* infection are still unavailable. Although some studies have found that Mp1p protein (Wang et al., 2011), galactomannan (GM) antigen (Li et al., 2020) or *T. marneffei* antigen (Prakit et al., 2016) can assist in diagnosing *T. marneffei* infection, these diagnosis determinants lack sensitivity or specificity or are useful preferentially in only HIV-positive patients. Therefore, it is essential to explore promising serum biomarkers to diagnose *T. marneffei* infection in HIV-negative patients.

Sphingolipids (SPLs) are a class of lipids with a long-chain sphingoid base backbone. SPLs possess diverse structures; moreover, they not only are essential structural components of cell membranes but also participate in many important signal transduction processes, such as the regulation of cell growth, differentiation, senescence and programmed cell death, mediating various biological functions of cells (Lahiri and Futerman, 2007; Merrill et al., 2007). Abnormalities in SPL metabolism have also been implicated in a variety of diseases, including inflammation, infectious diseases, heart disease, diabetes, neurodegenerative diseases, tumors (Watek et al., 2019; Saleem et al., 2020; Sukocheva et al., 2020), and several respiratory disorders, including chronic obstructive pulmonary disease (COPD) and cystic fibrosis (CF) (Petrache and Petrusca, 2013; Bowler et al., 2015; Lee et al., 2015; Becker et al., 2018). Moreover, the physiological and pathological roles of SPLs as pathogenetic elements or biomarkers of diseases were also reviewed in previous studies (Matanes et al., 2019; Iessi et al., 2020). Due to its high sensitivity, specificity and throughput, as well as being rapid and reliable, SPL analysis by liquid chromatography-mass spectrometry (LC-MS) has become the most powerful strategy (Becker et al., 2018; Claus and Graeler, 2020) and has been employed in discovery of biomarkers of various diseases. For instance, we discovered the characteristic SPLs that could be used as diagnostic biomarkers to differentiate different types of polycystic ovary syndrome (PCOS) in our previous study (Li et al., 2019).

The current study therefore utilized our well-established LC-MS method to explore the characteristic SPLs in HIV-negative patients with *T. marneffei* infection, aiming to identify promising serum biomarkers to diagnose HIV-negative patients with *T. marneffei* infection. Additionally, in order to evaluate the specificity of the biomarkers to *T. marneffei* infection, another clinical cohort of patients, including HIV-negative patients with pulmonary aspergillosis, viral pneumonia, or bacterial pneumonia, was also employed in this study for comparison.

## Material and Methods

### Study Participant Enrollment

The patients and healthy controls were recruited from the First Affiliated Hospital of Guangzhou Medical University. Patients enrolled in this study included patients suffering from *T. marneffei* infection, pulmonary aspergillosis, or bacterial or viral pneumonia, and they were admitted for hospitalization during sample collection.

The inclusion and exclusion criteria for *T. marneffei* infection were as follows. The inclusion criteria referred to the guidelines of diagnosis and management of invasive fungal disease (Donnelly et al., 2019): 1) age ≥ 18 years; 2) negative HIV test; 3) clinical or (and) imaging manifestations of *T. marneffei* infection; and 4) clinically confirmed *T. marneffei* infection *via* microbiological or pathological examination. Furthermore, clinically confirmed patients met any of the following criteria: 1) the specimen smears were stained with Rayne’s stain and observed under a microscope and the typical morphology was round or ovular, with distinct-septum fungi (often inside macrophages); 2) *T. marneffei* was isolated from specimen culture; or 3) histopathological examination found *T. marneffei*, with purulent granulomatous changes and a large number of mononuclear macrophages. On the other hand, the exclusion criteria included 1) age < 18 years; 2) positive HIV test; and 3) unwillingness to participate or unwillingness to provide signed informed consent.

Additionally, patients with pulmonary aspergillosis, those with bacterial or viral pneumonia and healthy controls also fulfilled the criteria of 1) age ≥ 18 years; 2) negative HIV test; and 3) provision of signed informed consent. The definitive diagnostic criteria of pulmonary aspergillosis referred to the guidelines of diagnosis and management of aspergillus diseases (Ullmann et al., 2018). The definitive diagnostic criteria of bacterial or viral pneumonia referred to the guidelines of diagnosis and treatment of community-acquired pneumonia (CAP) in adults in China (Cao et al., 2018). All healthy controls were without any documented pulmonary infectious, chronic or malignant diseases.

### Sample Collection and Processing

Most of the samples were collected during hospitalization before treatment, and a few samples were collected after treatment while the pathogen test was still positive. Participants’ serum samples were obtained in accordance with routine clinical operation guidelines, and blood collection was performed by a professional nurse. Blood was collected in a separator tube, allowed to clot at room temperature for 30 min and then centrifuged. Serum samples were then collected and stored at -80°C before SPL extraction.

### Sphingolipidomic Analysis

Serum SPLs were extracted with a three-step extraction protocol as previously described (Li et al., 2019), and each sample was prepared in duplicate. In brief, 20 μL of serum sample, 0.75 mL of chloroform (CHCl_3_)/methanol (MeOH) (1:2, v/v) solvent, and 10 μL of 2.5 μM SPL internal standard (Avanti Polar Lipids Inc, AL, USA) were transferred into a borosilicate glass tube. The mixture was sonicated at room temperature for 30 s, followed by incubation at 48°C for 12 h to extract total lipids. Then, phospholipids were degraded by mild alkaline hydrolysis with 75 μL of 1 M potassium hydroxide. After neutralization with acetic acid, the supernatant was stored in a new bottle, and the residue was successively extracted with 1 mL of CHCl_3_/MeOH (2:1, v/v) solvent and a mixed solvent comprised of 0.4 mL of CHCl_3_/MeOH (1:2, v/v), 1 mL of CHCl_3_ and 2 mL of water (H_2_O). Finally, the extract was dried under a nitrogen stream and reconstituted in 100 μL of MeOH for ultrahigh-performance liquid chromatography (UHPLC)-MS detection.

Serum SPL analysis was performed using our well-established UHPLC-MS approach (Wang et al., 2014). Chromatographic separation was conducted on an Agilent 1290 Infinity UHPLC system (Agilent, Santa Clara, CA, USA) with an Agilent Eclipse Plus C_18_ column (100 mm × 2.1 mm, 1.8 μm). The mobile phase was composed of (A) MeOH/H_2_O/formic acid (FA) (60:40:0.2, v/v/v) and (B) MeOH/IPA/FA (60:40:0.2, v/v/v), both containing 10 mM ammonium acetate. The quantitation of SPLs was carried out on an Agilent 6460 triple-quadrupole (QQQ) MS in multiple reaction monitoring (MRM) and positive mode.

### Statistical Analysis

The SPL quantitative data were acquired using Agilent MassHunter Quantitative Analysis B.09.00 software. A heatmap plot was constructed using Multi Experiment Viewer (MeV) software 4.9.0 (http://mev.tm4.org). SIMCA software version 15.0.2 (Sartorious Stedim Biotech, Umea, Sweden) was used for multivariate statistical analysis, in which orthogonal partial least squares discriminate analysis (OPLS-DA) was performed to identify the differentially expressed SPLs responsible for the discrimination among different groups. SPLs with variable importance in projection (VIP) values larger than 1.00 were considered potential diagnostic biomarkers. The R^2^Y and Q^2^Y values indicate the goodness of the fit of the OPLS-DA model, and cumulative values of R^2^Y and Q^2^Y close to 1 indicate an excellent model. All statistical analyses were performed using SPSS software version 26.0 (IBM Corp., Armonk, NY, USA) and GraphPad Prism software version 5.00 (GraphPad Software, La Jolla, CA, USA). Data that were normally distributed were compared using analysis of variance (ANOVA). All hypothesis testing was two-sided, and *P* < 0.05 was considered statistically significant. The performances of the potential diagnostic biomarkers were evaluated by using the receiver operating characteristic (ROC) curve, which was plotted by sensitivity (true positive rate) against 1-specificity (false positive rate) for all possible cutoff values. The area under the ROC curve (AUC) reflected the diagnostic efficacy of the potential biomarkers. The closer the AUC was to 1, the better the overall diagnostic performance of the biomarker.

## Results

### Characteristics of Study Participants

A total of 33 patients and 13 healthy controls (Control group) were enrolled. Among the 33 patients, 16 had a definite diagnosis of *T. marneffei* infection (TM group), all without fungemia, and 2 patients were simultaneously infected with *Staphylococcus aureus*; 10 were confirmed to have pulmonary aspergillosis (PA group), and all had chronic pulmonary aspergillosis; 2 were diagnosed with viral pneumonia (VP group), and the pathogen was identified as an influenza virus; and 5 patients had bacterial pneumonia (BP group), and although the pathogen was unclear, it was confirmed to be unrelated to *T. marneffei*, aspergillosis, or influenza virus infection. The characteristics of the participants are shown in **Table 1**. The BP group showed a significant difference in age with the Control (*P* = 0.0062), TM (*P* = 0.0106), and PA groups (*P* = 0.0141), while no significant difference in age or sex was observed among the other groups.

**Table 1:** Characteristics of healthy controls (Control), and patients with *T. marneffei* infection (TM), bacterial pneumonia (BP), viral pneumonia (VP), and pulmonary aspergillosis (PA).

Group	Number of cases (*n*)	Age, years*^a^*	Gender (female), %
**Control**	13	38.92 (31.32-46.53)	53.85
**TM**	16	42.75 (34.67-50.83)	68.75
**BP**	5	71.00 (51.51-90.49)*^b^*	60.00
**VP**	2	50.50 (0.50-101.50)	100.00
**PA**	10	44.40 (35.57-53.23)	50.00

a Data are presented as the mean and (95% confidence interval).

b BP show significant difference in age with Control (P = 0.0062), TM (P = 0.0106), and PA (P = 0.0141), and no significant difference in age and gender are observed within other group.

### Quantitation of Targeted Serum SPLs

**Figure 1:**
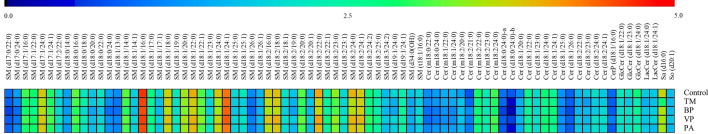
Heat map showing level of each serum sphingolipid (SPL) in healthy controls (Control) (*n* = 26), patients with *T. marneffei* infection (TM) (*n* = 32), patients with bacterial pneumonia (BP) (*n* = 10), patients with viral pneumonia (VP) (*n* = 4), and patients with pulmonary aspergillosis (PA) (*n* = 20). For each SPL, the mean concentrations within different groups were log 10 transformed. The color legend ranges from blue to red indicating low to high level.

Through our well-established UHPLC-QQQ-MS approach, 80 SPLs were quantified from each sample, including 50 sphingomyelins (SMs), 22 ceramides (Cers), 3 hexosylceramides (HexCers), 2 lactosylceramides (LacCers), 1 sphingosine (So), 1 sphinganine (Sa), and 1 ceramide-1-phosphate (Cer1P). The level of each SPL among the Control, TM, BP, VP, and PA groups was visualized using a heatmap plot (**Figure 1**). The heatmap plot revealed that SM was the most abundant species of serum SPLs in all groups, in which d18:1 was the dominant type and SM (d18:1/16:0) was the most abundant species.

### Differences in Serum SPLs Between Each Group of Subjects

**Figure 2:**
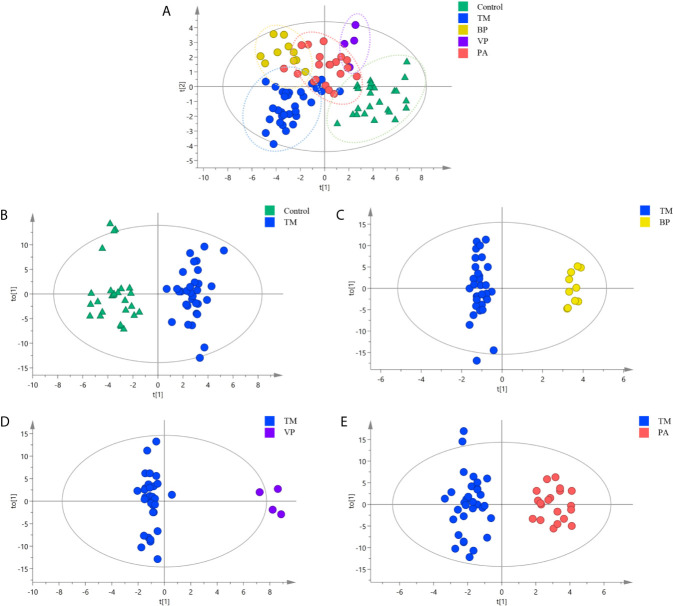
Orthogonal partial least squares discriminate analysis (OPLS-DA) score plots for discriminating **(A)** all the sample groups, including healthy controls (Control group) (*n* = 26), and patients with *T. marneffei* infection (TM group) (*n* = 32), with bacterial pneumonia infection (BP group) (*n* = 10), with viral pneumonia infection (VP group) (*n* = 4), and with pulmonary aspergillosis infection (PA group) (*n* = 20) (R^2^X = 0.869, R^2^Y = 0.679, Q^2^ = 0.455); **(B)** TM group and Control group (R^2^X = 0.805, R^2^Y = 0.909, Q^2^ = 767); **(C)** TM group and BP group (R^2^X = 0.836, R^2^Y = 0.981, Q^2^ = 0.881); **(D)** TM group and VP group (R^2^X = 0.804, R^2^Y = 0.973, Q^2^ = 0.836); and **(E)** TM group and PA group (R^2^X = 0.769, R^2^Y = 0.926, Q^2^ = 0.659).

Multivariate analysis was then conducted to explore the SPLs associated with discrimination among different sample groups. As shown in **Figure 2A**, the Control group displayed obvious separation with those infection groups, while certain overlaps were existed among different infection groups (R^2^X = 0.869, R^2^Y = 0.679, Q^2^ = 0.455). This revealed that the Control group possessed distinct different serum SPLs when compared with those infection groups, whereas certain similarities in serum SPLs were present among different infection groups.

**Figure 3:**
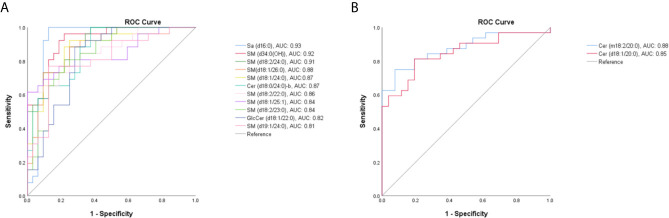
The receiver operating characteristic (ROC) curve for SPLs with an area under the ROC curve (AUC) value > 0.80. **(A)** Eleven declined SPLs and **(B)** 2 elevated SPLs after *T. marneffei* infection.

Then, the serum SPLs in the TM group was further compared with other groups, respectively. As shown in **Figure 2B**, the OPLS-DA score plot demonstrated a clear separation between the TM group and the Control group (R^2^X = 0.805, R^2^Y = 0.909, Q^2^ = 0.767), and 32 SPLs (24 SMs, 5 Cers, 2 GlcCers, and 1 Sa) were identified as potential diagnostic biomarkers for *T. marneffei* infection. Among them, 13 SPLs displayed AUCs > 0.80, including 11 declined SPLs (**Figure 3A**) and 2 elevated SPLs (**Figure 3B**). In particular, Sa (d16:0) with an AUC of 0.928 (95% confidence interval [CI]: 0.848-1.000), SM [d34:0(OH)] with an AUC of 0.922 (95% CI: 0.856-0.988), and SM (d18:2/24:0) with an AUC of 0.905 (95% CI: 0.833-0.977) were the most promising SPL biomarkers to discriminate between patients suffering from *T. marneffei* infection and healthy controls.

**Figure 4:**
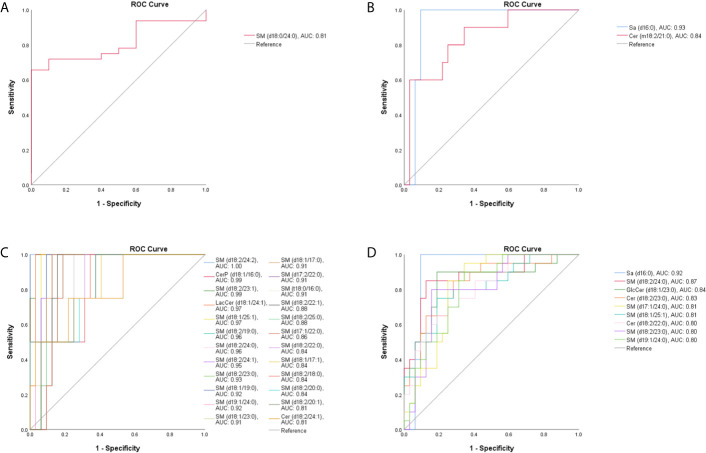
The ROC curve for SPLs with an AUC value > 0.80. **(A)** One SPL displayed a higher level and **(B)** 2 SPLs displayed lower levels in patients with *T marneffei* infection (TM group) than in patients with bacterial pneumonia. **(C)** Twenty-four SPLs capable of differentiating TM subjects from patients with viral pneumonia. **(D)** Nine SPLs that could distinguish TM subjects from patients with pulmonary aspergillosis.

OPLS-DA was also conducted to differentiate *T. marneffei* infection from infections caused by other microorganisms. The OPLS-DA score plot in **Figure 2C** displays a distinct separation between the TM and the BP groups (R^2^X = 0.836, R^2^Y = 0.981, Q^2^ = 0.881), and 28 SPLs (15 SMs, 9 Cers, 2 LacCers, 1 GlcCers, and 1 Sa) were discovered as potential biomarkers to distinguish *T. marneffei* infection from bacterial infection. Only 3 SPLs possessed AUCs > 0.80, including SM (d18:0/24:0) with an AUC of 0.809 (95% CI: 0.681-0.938), which displayed a higher level in the TM group (**Figure 4A**), and Sa (d16:0) with an AUC of 0.925 (95% CI: 0.838-1.000), as well as Cer (m18:2/21:0) with an AUC of 0.841 (95% CI: 0.703-0.978), which showed a lower level in the TM group (**Figure 4B**). Of note, Sa (d16:0) possessed the highest AUC and was therefore regarded as the most significant SPL biomarker to distinguish the TM group from the BP group.

Moreover, as illustrated in **Figures 2D** **, E**, the OPLS-DA score plots demonstrate a clear separation between the TM group and the VP group (R^2^X = 0.804, R^2^Y = 0.973, Q^2^ = 0.836) and between the TM group and the PA group (R^2^X = 0.769, R^2^Y = 0.926, Q^2^ = 0.659). Moreover, 29 SPLs (23 SMs, 4 Cers, 1 LacCer, and 1 CerP) and 26 SPLs (17 SMs, 6 Cers, 1 GlcCer, 1 LacCer, and 1 Sa) were found to be potential biomarkers to differentiate *T. marneffei* infection from viral infection and aspergillosis infection, respectively. As shown in **Figure 4C**, 24 out of the 29 potential SPL biomarkers capable of differentiating the TM group from the VP group displayed AUCs > 0.80, including SM (d18:2/24:2) with AUC of 1.00 and both CerP (d18:1/16:0) and SM (d18:2/23:1) with AUCs of 0.992 (95% CI: 0.968-1.000). Notably, all these SPLs showed higher levels in the VP group. In addition, 9 out of the 26 potential SPL biomarkers that could distinguish the TM group from the PA group illustrated an AUC > 0.80 (**Figure 4D**), and Sa (d16:0) with an AUC of 0.919 (95% CI: 0.828-1.000) was the most obvious biomarker. All these SPLs showed higher levels in the PA group.

### Potential Serum SPL Diagnostic Biomarkers Specific for *T. marneffei* Infection

**Figure 5:**
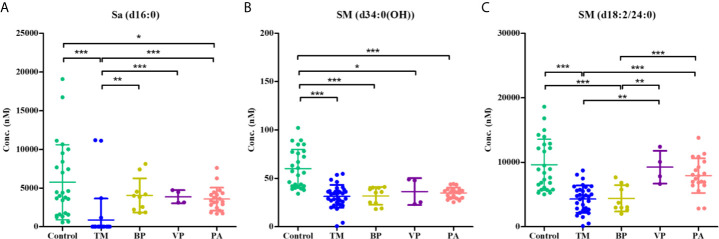
Scatter plots showing the serum levels of **(A)** Sa (d16:0), **(B)** SM [d34:0(OH)], and **(C)** SM (d18:2/24:0) within the control group (*n* = 26), the TM group (*n* = 32), the BP group (*n* = 10), the VP group (*n* = 4), and the PA group. Horizontal lines indicate the mean ± SD (*P < 0.05, **P < 0.01, ***P < 0.001).

Sa (d16:0), SM [d34:0(OH)], and SM (d18:2/24:0) were identified as the most promising SPL biomarkers capable of discriminating between patients with *T. marneffei* infection and healthy controls. The serum levels of these 3 SPLs among each group of subjects are illustrated in **Figure 5**. As shown in **Figure 5A**, there was an overall phenomenon of obvious decline of Sa (d16:0) after infection with microorganisms. Notably, the level of Sa (d16:0) in the TM group was significantly lower than that in the BP, VP, and PA groups. Therefore, Sa (d16:0) was considered a specific diagnostic biomarker for *T. marneffei* infection, and 302.71 nM was selected as the optimal cutoff value with a diagnostic sensitivity of 87.50% and specificity of 100.00%. As shown in **Table 2**, of the 32 TM samples, 28/32 (87.50%) had a Sa (d16:0) concentration < 302.71 nM, while none of the samples from the Control or other groups infected with other microorganisms exhibited Sa (d16:0) concentrations < 302.71 nM. Therefore, serum concentrations of Sa (d16:0) less than 302.71 nM were considered specific diagnostic biomarkers of *T. marneffei* infection. Similarly, the levels of SM [d34:0(OH)] and SM (d18:2/24:0) universally declined after infection with microorganisms. However, there were no significant discrepancies in SM [d34:0(OH)] levels among the groups infected with different microorganisms (**Figure 5B**). Moreover, the level of SM (d18:2/24:0) in the TM group showed no obvious differences from that in the BP group, although it was significantly lower than that in the VP and PA groups (**Figure 5C**).

**Table 2:** Serum concentration of Sa (d16:0) in healthy controls, and patients with *T. marneffei* infection (TM), bacterial pneumonia (BP), viral pneumonia (VP), and pulmonary aspergillosis (PA).

Group	Sa (d16:0) concentration (nM)		Number (% with concentration of Sa (d16:0) < 302.71 nM)
	95% confidence interval	Mean	
**Control (*n* = 26)**	3802.35-7714.98	5758.67	0 (0.00)
**TM (*n* = 32)**	-96.20-1830.17	866.99	28 (87.50)
**BP (*n* = 10)**	2479.26-5625.42	4052.34	0 (0.00)
**VP (*n* = 4)**	3209.36-4530.04	3869.70	0 (0.00)
**PA (*n* = 20)**	2878.59-4265.66	3572.13	0 (0.00)

## Discussion

Recently, more attention has been directed to *T. marneffei* infection due to its high mortality rate. The fatality rate of HIV-negative patients is even higher than that of HIV patients as a result of diagnostic delay due to the lack of clinical suspicion (Chan et al., 2016). Therefore, early diagnosis is critical to reduce the fatality rate. However, nonspecific symptoms and biological characteristics similar to those of other common pathogenic fungi make the rapid and accurate diagnosis of *T. marneffei* infection challenging (Rinaldi, 1996; Mootsikapun and Srikulbutr, 2006). Microscopic examination and mycological culture are the traditional and gold standard methods for the diagnosis of *T. marneffei* infection (Cao et al., 2019). Nevertheless, microscopic examinations involve invasive operations, such as bone marrow aspirate collection and skin or lymph node biopsy. Moreover, mycological cultures are time consuming and take up to 14 days, and the diagnostic sensitivity of different samples varies; the sensitivity of blood culture is only 76% (Supparatpinyo et al., 1994).

In recent decades, non-culture-based approaches have been developed for the rapid diagnosis of *T. marneffei* infection. GM is a heteropolysaccharide present in the cell walls of most *Aspergillus* and *Talaromyces* species, and the GM assay was identified as a useful tool to diagnose *T. marneffei* infection in HIV patients (Huang et al., 2007; Becker et al., 2018). A subsequent study discovered that the diagnostic sensitivity of the GM assay in HIV patients (100%) was superior to that in HIV-negative patients (68.18%), whereas the diagnostic sensitivity in HIV-negative patients without fungemia was only 57.14% (Li et al., 2020). Moreover, previous studies have reported using polymerase chain reaction (PCR)-based assays that target the ribosomal DNA or *MP1* gene of *T. marneffei* to identify *T. marneffei* in clinical samples, including whole blood, plasma, serum, or paraffin-embedded tissues (Zeng et al., 2009; Zhang et al., 2011; Hien et al., 2016; Lu et al., 2016; Li et al., 2020). The diagnostic sensitivity of assays using paraffin-embedded tissues reached 100% (Zeng et al., 2009; Zhang et al., 2011). However, PCR methods require high-quality DNA samples that are difficult to obtain from patients’ blood samples, which is why the diagnostic sensitivity using blood samples is generally < 80% (Hien et al., 2016; Lu et al., 2016), and the diagnostic sensitivity for HIV-negative patients without fungemia is only 64.29% (Li et al., 2020). Furthermore, several diagnostic approaches based on antibody or antigen detection have been developed (Wang et al., 2011; Wang et al., 2015; Prakit et al., 2016; Li et al., 2019; Ly et al., 2020; Thu et al., 2020). A recent study employing a *T. marneffei*-specific mannoprotein (Mp1p) antigen-detecting enzyme immunoassay exhibited higher diagnostic sensitivity for *T. marneffei* infection than both a PCR assay (86.3% vs 70-80%) and standard BACTEC blood culture (86.3% vs 72.8%) (Thu et al., 2020). However, these studies did not specify whether the patients were HIV positive or HIV negative, and these methods required the physician to suspect the pathogen before examination, which might restrict its clinical application (Thu et al., 2020). In addition, a case report describing the diagnosis of *T. marneffei* infection in an HIV-negative patient with the assistance of the next-generation sequencing (NGS) technique has been published; however, NGS is limited in differentiating among colonization, infection, and contamination, and other laboratory tests are still needed to confirm the causative pathogen (Zhu et al., 2018).

There are varied limitations of the previous methods, and the study objects of these methods are generally HIV positive or of unknown status. Therefore, we propose an alternative diagnostic method specific for *T. marneffei* infection in HIV-negative patients in this study. Through a sphingolipidomic approach and multivariate analysis, three serum SPLs, Sa (d16:0), SM [d34:0(OH)], and SM (d18:2/24:0), were identified as the most promising biomarkers to discriminate between healthy controls and patients with *T. marneffei* infection. Among them, Sa (d16:0) was considered a specific diagnostic biomarker for *T. marneffei* infection, and 302.71 nM was selected as the optimal cutoff value with a diagnostic sensitivity of 87.50% and specificity of 100.00%. LC-MS has been increasingly used in routine clinical laboratories during the last two decades (Grebe and Singh, 2011; Leung and Fong, 2014). Therefore, this finding could help to develop a simple LC-MS assay in clinical laboratories that employing a calibration curve strategy to quantify the level of Sa (d16:0) in each sample within several minutes. Notably, 4 out of the 32 TM samples had a Sa (d16:0) concentration higher than 302.71 nM. These 4 samples were derived from two patients, and their average concentrations of duplicate samples were 11150.60 nM and 2429.52 nM. In contrast, the serum concentration of Sa (d16:0) in other patients was lower than 50 nM. Retrospective review of the medical records of the two patients illustrated that they were concomitantly infected with *Staphylococcus aureus*; therefore, they displayed extraordinarily higher serum concentrations of Sa (d16:0) than other TM patients. Hence, this condition revealed one of the limitations of our study: Sa (d16:0) is applicable only to screen TM patients who are infected only with *T. marneffei*, and this biomarker is unable to identify TM patients who are concomitantly infected with other pathogens. In addition, this study had other limitations. For instance, a small sample size limited the reliability of the biomarker’s diagnostic power; therefore, a larger cohort of patients with *T. marneffei* infection and other fungal diseases is necessary for further evaluation of the actual sensitivity and specificity of using Sa (d16:0) as a specific diagnostic biomarker for *T. marneffei* infection. Moreover, this study is a retrospective case-control study for which estimation of diagnostic accuracy was available only when the diagnosis was already known; therefore, prospective diagnostic studies are needed to screen patient populations who are at risk for *T. marneffei* infection. On the other hand, although most of the samples were collected during hospitalization before treatment, and a few samples were collected after treatment while the pathogen test was still positive, there were no differences between them in Sa (d16:0) level.

Most bacteria and viruses do not produce SPLs but utilize host SPLs for survival and to promote their virulence. On the other hand, some bacteria and fungi can produce SPLs, which consequently leads to both host and pathogen SPLs being involved in microbial pathogenesis (Heung et al., 2006). Based on the rapid, reliable, and high sensitivity, specificity, and throughput properties of LC-MS, it is possible to characterize the sphingolipidome of large-scale clinical samples, resulting in a systemic overview of the dynamic host-parasite interaction and recognition of the SPLs that mediate pathological processes (Lee et al., 2014; Chong et al., 2018; Thu et al., 2020). Several studies have reviewed the important role of SPLs in the progression of infection (Hanada, 2005; Maceyka and Spiegel, 2014; Chong et al., 2018; Li et al., 2019; Claus and Graeler, 2020). For instance, elevated plasma levels of SM (d18:0/16:0), SM (d18:1/16:0) and three glycosphingolipids were observed in CAP patients (To et al., 2016). In contrast, a 5-fold decrease in SM (d18:1/16:0) content along with increases in total Cer content and lymphocyte counts were observed in patients with dengue fever at an early febrile stage (Cui et al., 2013). In addition, a combination of SM (d18:1/22:3) with glycerophospholipids exhibited promising diagnostic sensitivity for sepsis (Neugebauer et al., 2016). A recent study reported that 3 out of the top 10 metabolites that were able to distinguish healthy controls from COVID-19 patients were SPLs, as plasma levels of SM (d18:1/18:1) and another acidic glycosphingolipid were elevated in COVID-19 patients. Conversely, the plasma level of S1P (d18:1) was decreased in COVID-19 patients and exhibited strong predictive value for prognosis (Song et al., 2020). More importantly, numerous studies have demonstrated that sphingosine has remarkable antibacterial activity against a variety of pathogens and is a pivotal first-line defense in healthy airways; its level is greatly reduced in the airways of patients and mice with CF (Pewzner-Jung et al., 2014; Grassme et al., 2017; Martin et al., 2017; Becker et al., 2018). These findings might help to explain why the level of Sa (d16:0) was generally decreased after infection with different microorganisms in our current study.

In summary, our study proposes an alternative diagnostic method specific for *T. marneffei* infection in HIV-negative patients by employing Sa (d16:0) as a promising diagnostic biomarker, and 302.71 nM was selected as the optimal cutoff value with a diagnostic sensitivity of 87.50% and specificity of 100.00%. However, this biomarker is unable to identify TM patients who are concomitantly infected with other pathogens, and a larger cohort of patients is needed to validate the diagnostic power of Sa (d16:0) for *T. marneffei* infection.

## Data Availability Statement

The raw data supporting the conclusions of this article will be made available by the authors, without undue reservation.

## Ethics Statement

The studies involving human participants were reviewed and approved by the Ethics Committee of the First Affiliated Hospital of Guangzhou Medical University (ethical approval number: 2019-26). The patients/participants provided their written informed consent to participate in this study.

## Author Contributions

Z-TL, L-FY, J-RW, and FY designed the study and drafted the manuscript. Z-TL, YQ, S-QL, Y-QZ, and J-QZ conducted patient recruitment, enrollment and specimen collection. YL and JC registered patient information. Z-MC, YML, and ZL handled clinical specimen and storage. L-FY, W-HC, Z-HJ, and J-RW performed statistical analysis and data interpretation. All authors contributed to the article and approved the submitted version.

## Funding

This research was funded by the Independent Fund of the State Key Laboratory of Respiratory Diseases (SKLRD-OP-201913; SKLRD-Z-202019); the Guangzhou Institute of Respiratory Health Open Project (2019GIRHZ06); and the Science and Technology Development Fund, Macau SAR (file no. 082/2017/A2 to J-RW).

## Conflict of Interest

The authors declare that the research was conducted in the absence of any commercial or financial relationships that could be construed as a potential conflict of interest.
